# Adherence to etanercept therapy in rheumatoid arthritis patients during 3 years of follow-up

**DOI:** 10.1371/journal.pone.0205125

**Published:** 2018-10-09

**Authors:** E. H. Vogelzang, R. C. F. Hebing, M. T. Nurmohamed, A. W. R. van Kuijk, J. W. F. Kruijff, M. J. l’Ami, C. L. M. Krieckaert, G. Wolbink

**Affiliations:** 1 Amsterdam Rheumatology and Immunology Center | Reade, Rheumatology, Amsterdam, The Netherlands; 2 Amsterdam Rheumatology and Immunology Center | Reade, Rheumatology, Pharmacy, Amsterdam, The Netherlands; 3 University of Amsterdam, Faculty of Science, Amsterdam, The Netherlands; 4 Sanquin Research and Landsteiner Laboratory, Immunopathology, Amsterdam, The Netherlands; Auburn University College of Veterinary Medicine, UNITED STATES

## Abstract

**Objectives:**

To determine the percentage non-adherence to etanercept in patients with rheumatoid arthritis during three years of follow-up.

**Methods:**

During study visits in this prospective cohort study, blood samples were taken to determine serum etanercept concentrations using ELISA and patients were asked if they had missed an etanercept dose, at which date and for what reason. Non-adherence was defined as serum etanercept concentration <0.1 μg/mL and no valid reason to miss the prescribed etanercept dose.

**Results:**

In total, 292 consecutive patients treated with etanercept were included. Most patients had a valid reason to miss their etanercept dose (25/37). In total 12 out of 292 patients (4.1%, 95% confidence interval 2.2–7.2) were non-adherent during the 3 year period. In a small percentage of patients (3.4%, 95% confidence interval 0.8–10.4) who failed to respond to etanercept therapy, according to their rheumatologist, this was associated with inadequate exposure to etanercept and thus non-adherence.

**Conclusion:**

In this study, adherence to etanercept therapy was measured using serum etanercept concentration. In most patients an absent etanercept concentration was due to a medical reason. Furthermore, the majority of patients were adherent to etanercept therapy and had adequate drug exposure. In total, only 12 out of 292 patients (4.1%) were non-adherent during 3 years of follow-up. These findings highlight that only a small minority of patients are non-adherent to etanercept treatment, especially compared to adherence rates of other drugs. However, physicians should be aware that in patients failing to respond to treatment, non-adherence is a possible cause.

## Introduction

The question: “How good is patient adherence to biological agents and is lack of adherence related to loss of efficacy?” was part of the research agenda that was formulated in the European League Against Rheumatism’s (EULAR) recommendations for the management of rheumatoid arthritis (RA) with synthetic and biological disease-modifying anti rheumatic drugs (respectively sDMARDs and bDMARDs) [1]. A concern is that, without adherence, optimal benefits of the prescribed medication will not be achieved and could influence clinical outcome, medication costs and health care utilization. Furthermore, rheumatologists could erroneously conclude that patients failed to respond to therapy when there was no adequate exposure to the prescribed treatment.

Limited studies are published in which adherence of RA patients treated with bDMARDs have been assessed. For RA patients treated with bDMARDs, mainly etanercept and adalimumab have been studied. The percentage of patients adherent to bDMARDs varies from 32% to 90% [2–11]. Predominantly this has been measured using medication possession ratios (MPR).

The World Health Organisation (WHO) has defined adherence as “the extent to which a person’s behaviour–taking medication, following a diet, and/or executing lifestyle changes, corresponds with agreed recommendations from a health care provider” [12]. The WHO definition of adherence is particularly relevant for RA patients being treated with bDMARDs since they are instructed to temporarily discontinue bDMARDs therapy during e.g. an operation or a serious infection. Therefore, temporarily discontinuing bDMARDs therapy, e.g. during a serious infection or at time of surgery does not constitute non-adherence. Previous studies did not take this into account when assessing non-adherence in RA patients treated with bDMARDs [2–6, 8–11]. Furthermore, there are several methods to detect adherence. However the least biased method to assess adherence is measuring drug serum concentrations [13]. To our knowledge, serum concentrations of bDMARDs have not been used in assessing adherence to bDMARD in RA patients, since factors such as immunogenicity could account for absent drug concentrations. Most studies, however, have not detected anti-drug antibodies against etanercept, making it an ideal drug for which to use serum concentration to determine non-adherence [14, 15].

The aim of the study is to assess the percentage of non-adherence, thereby indicating whether non-adherence is a major concern when treating RA patients with etanercept.

The current study describes the percentage non-adherence to etanercept therapy, using measurement of serum etanercept concentrations and patient self-report during three years of follow-up.

## Materials and methods

### Participants

This observational prospective cohort study was conducted at the Amsterdam Rheumatology and Immunology Center | Reade, Amsterdam, The Netherlands. In this cohort study, consecutive RA patients, aged 18 years or older, starting etanercept treatment were included. These patients have been partially described before [14]. All patients were diagnosed with RA according to the American College of Rheumatology 1987 revised criteria [16]. Before starting etanercept, patients failed to respond adequately to at least two sDMARDs. The decision to start with etanercept was at the discretion of the treating rheumatologist. Patients were treated with either etanercept 50mg once weekly or 25mg twice per week based on the patient’s preference. Patients were treated either with etanercept monotherapy, etanercept with concomitant sDMARDs and with or without prednisone, reflecting routine clinical practice.

### Adherence

Non-adherence was defined as serum etanercept trough concentration below the lowest level of detection (<0.1ug/mL) at least once and no valid/medical reason (e.g. tapering or an infection) to miss an etanercept dose. Based on the half-life of etanercept (approximately 70 hours) a serum etanercept concentration below 0.1ug/mL indicates that at least one previous dose was missed before blood was drawn.

#### Measuring etanercept concentrations

To measure etanercept levels maxisorp ELISA plates were coated overnight at room temperature with 2 μg/mL monoclonal anti-TNF-5 (Sanquin, Amsterdam) in PBS. After five times washing with PBS/0.02% Tween 20 (PT), plates were incubated for 1 hour at room temperature with recombinant TNFα (0.05 μg/mL) (Strathmann Biotech GmbH, Hannover, Germany) diluted in high performance ELISA buffer (HPE, Business Unit reagents, Sanquin, Amsterdam). Next, the plates were washed and incubated for 1 hour with patient serum which was serially diluted in HPE. Subsequently, the plates were washed with PT and incubated for 1 hour with biotinylated etanercept specific rabbit anti-idiotype (0.5 μg/mL in HPE). After washing streptavidin-poly-HRP (Sanquin) (1/25000, in HPE) was added for 15 minutes at 30°C. After washing the ELISA was developed with 100 μg/mL tetramethylbenzidine in 0.11 M sodium acetate (pH 5.5) containing 0.003% (v/v) H2O2. The reaction was stopped with 2 M H2SO4. Absorption at 450 nm was measured. Results were related to a titration curve of etanercept in each plate. The lowest level of detection was 0.1 μg/mL.

#### Patient self-report

To ascertain self-reported adherence, patients were asked during each visit, “Did you missed any doses of etanercept since the last time you visited our outpatient clinic? If so, when and for what reason?”. This was recorded in the patient record. For all patients with serum etanercept concentrations under 0.1 μg/mL, we consulted the patient’s file for the potential reasons for the missed dose(s).

### Clinical and demographical data

Patient visits were planned at baseline and at 4, 16, 28, 40, 52, 78, 104, 130 and 156 weeks after initiation of treatment with etanercept. Demographical data included age and gender. At baseline, information regarding DMARD therapy such as previous number of DMARDs, current DMARD use and current methotrexate dose were collected. Disease-related parameters collected at baseline included disease duration, 28 joint disease activity score, C-reactive protein, erythrocyte sedimentation rate and rheumatoid factor. Blood samples were drawn at baseline and at subsequent visits.

### Statistical analysis

To assess significant differences between groups at baseline, we used the independent sample t test χ^2^ or Mann-Whitney U test, as appropriate.

Statistical tests were 2-sided, if appropriate, and a p-value <0.05 was considered significant. Statistical analysis was performed using SPSS version 19 for Windows (SPSS Inc., Chicago, Illinois). To calculate the percentage of adherent patients, the incidence rate and the 95% confidence interval software R version 3.4.2 for Windows was used. All graphs were made using GraphPad Prism version 6 (GraphPad Software, Inc, San Diego, California).

### Ethical clearance

Approval for this study was obtained from the medical ethics committee of Slotervaart Hospital and the Jan van Breemen Research Institute | Reade. With agreement from the patient, the treating rheumatologist referred eligible patients. Subsequently, patients were invited to visit the clinic, during which, information about the cohort study was provided and written informed consent obtained. All patients gave their written informed consent.

## Results

### Characteristics of study population and follow-up

A total of 292 RA patients who started treatment with etanercept were included. The median age was 54 years and the majority of patients were female (82%, see Table 1). Non-adherent patients were significantly younger (median age 43 years, p = 0.041) and had previously received significantly less DMARDs compared to adherent patients. In Table 1 the patient’s baseline characteristics are shown in detail.

**Table 1:** Baseline characteristics.

	Total number of patients (n = 292)	Adherent patients (n = 280)	Non-adherent patients (n = 12)
Age years, median (IQR)	54 (43–62)	54 (43–62)*	43 (37–53)*
Female, n(%)	239 (82)	229 (82)	10 (83)
BMI, median (IQR)	25 (22–29)	25 (22–29)	21 (21–34)
Previous DMARD, median (IQR)	3 (2–4)	3 (2–4)*	2 (2–3)*
MTX use, n (%)	223 (76)	214 (76)	9 (75)
MTX dose, mg/week, median (IQR)	22.5 (15.0–25.0)	23.8 (15.0–25.0)	22.5 (10.0–25.0)
Prednisone use, n (%)	83 (28)	78 (28)	5 (42)
Prednisone dose mg/day, median (IQR)	7.5 (5.0–10.0)	7.5 (5.0–10.0)	10 (3.8–12.5)
Other DMARD than MTX, n (%)	96 (33)	91 (33)	5 (42)
Previous biological, n (%)	89 (31)	86 (31)	3 (25)
Disease duration (years), median (IQR)	8 (3–16)	8 (3–16)	6 (2–14)
Rheumatoid factor, n (%)	208 (71)	201 (72)	7 (58)
Erosive disease, n (%)	207 (71)	199 (71)	8 (67)
HAQ, median (IQR)	1.3 (0.8–1.8)	1.3 (0.8–1.8)	1.3 (1.2–1.7)
DAS28, median (IQR)	5.3 (4.4–6.0)	5.3 (4.2–6.0)	4.8 (4.6–5.4)
ESR, mm/h, median (IQR)	23 (12–40)	23 (12–40)	15 (7–25)
CRP, mg/L, median (IQR)	8 (3–21)	8 (3–21)	6 (2–10)

BMI, body mass index; CRP, C-reactive protein; DAS28, 28 joint disease activity score; DMARD, disease-modifying antirheumatic drugs; ESR, erythrocyte sedimentation rate; HAQ, health assessment questionnaire; MTX, methotrexate.

*A significant difference was found for: age; p = 0.041 and previous DMARD; p = 0.033.

During three years of follow-up, 141 patients (48.3%) dropped out of the study. Of these 87 (62%) dropped out due to treatment failure, 24 patients (17%) due to adverse events and 30 patients (21%) due to various reasons such as lost-to-follow-up or relocation.

### Percentage of non-adherent patients

**Fig 1:**
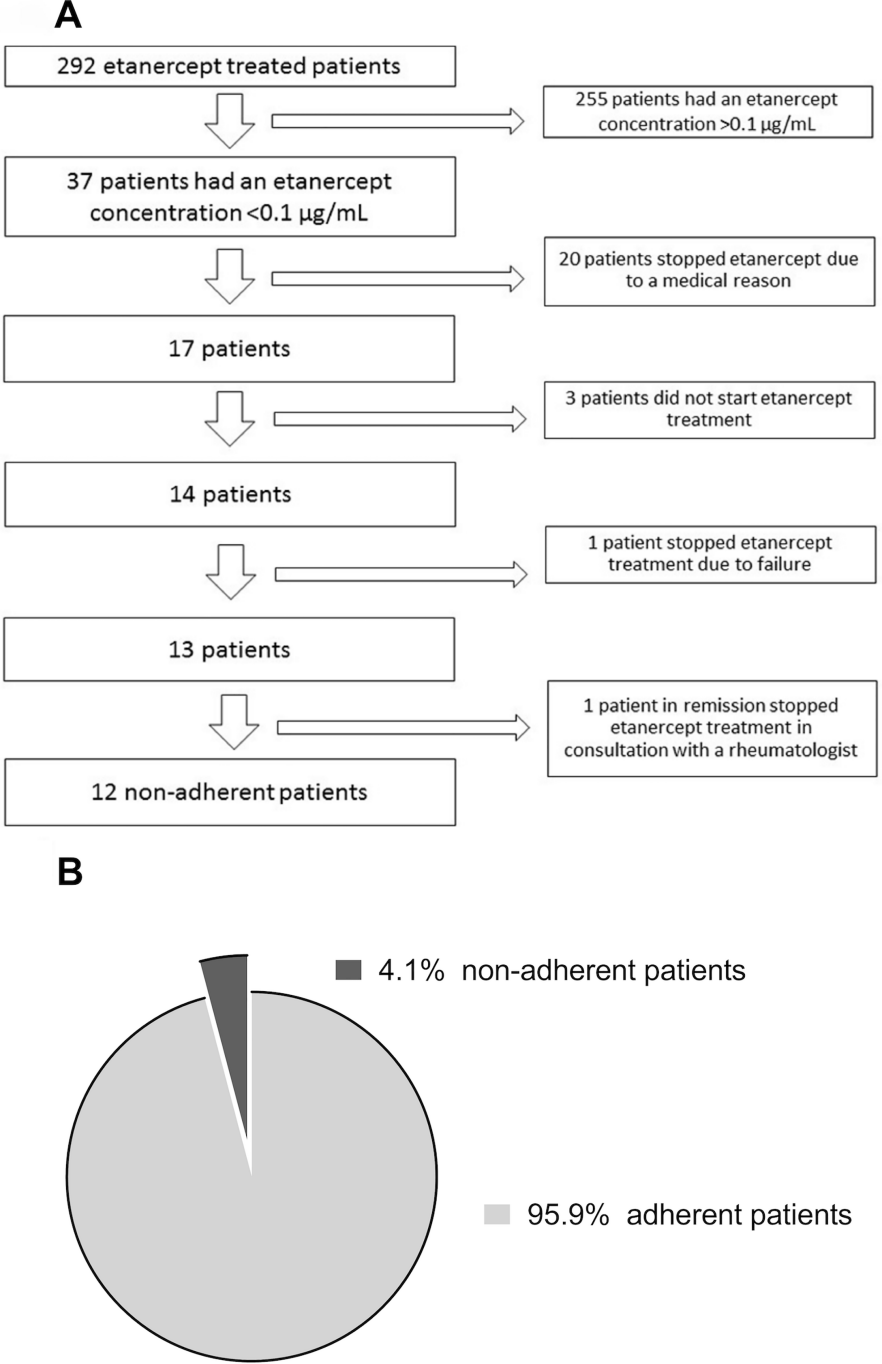
**A** The flow chart of the study population. **B** Percentage of patients who were non-adherent over time (n = 292).

All baseline serum samples obtained before initiation of etanercept therapy were negative for etanercept. The median etanercept concentration for all visits (except baseline) was 3.2 μg/mL (2.12–4.54). During follow-up, 37 patients had a serum etanercept concentration <0.1 μg at least once. Fig 1A shows the reasons patients missed an etanercept dose. Most patients (20/37) had a medical reason to miss an etanercept dose, mostly infections. See Table 2 for the different medical reasons.

**Table 2:** Medical reasons for patients who missed an etanercept dose.

Number of patients (n = 20)	Medical reason
6	Respiratory tract infection
2	Side-effects
2	Pregnancy
1	Interstitial lung disease
1	Urinary tract infection
1	Cellulitis
1	Gastro-enteritis
1	Recurrent infections
1	Breast cancer
1	Operation
1	Infection after osteosynthesis
1	Infection of the mouth
1	Infected abscess

In total, 12 patients (4.1%, 95% confidence interval [95% CI] 2.2–7.2) were non-adherent during follow-up of three years (see Fig 1B; in weeks 4, 16, 28, 40, 52 and 156 there were respectively 3, 3, 1, 1, 2 and 2 patient[s] non-adherent). In total, 10 out of 12 patients were non-adherent during the first year. The median time at which patients were non-adherent was 22 weeks (IQR 4–52). The incidence rate was 22.4/1000 person-years (95% CI 14.5–34.2).

In the majority of non-adherent patients, there was no record of missing an etanercept dose. Only one non-adherent patient reported not taking an etanercept dose. In total, four non-adherent patients had an etanercept concentration <0.1μg/mL at least twice.

### Influence of non-adherence on clinical outcome

**Fig 2:**
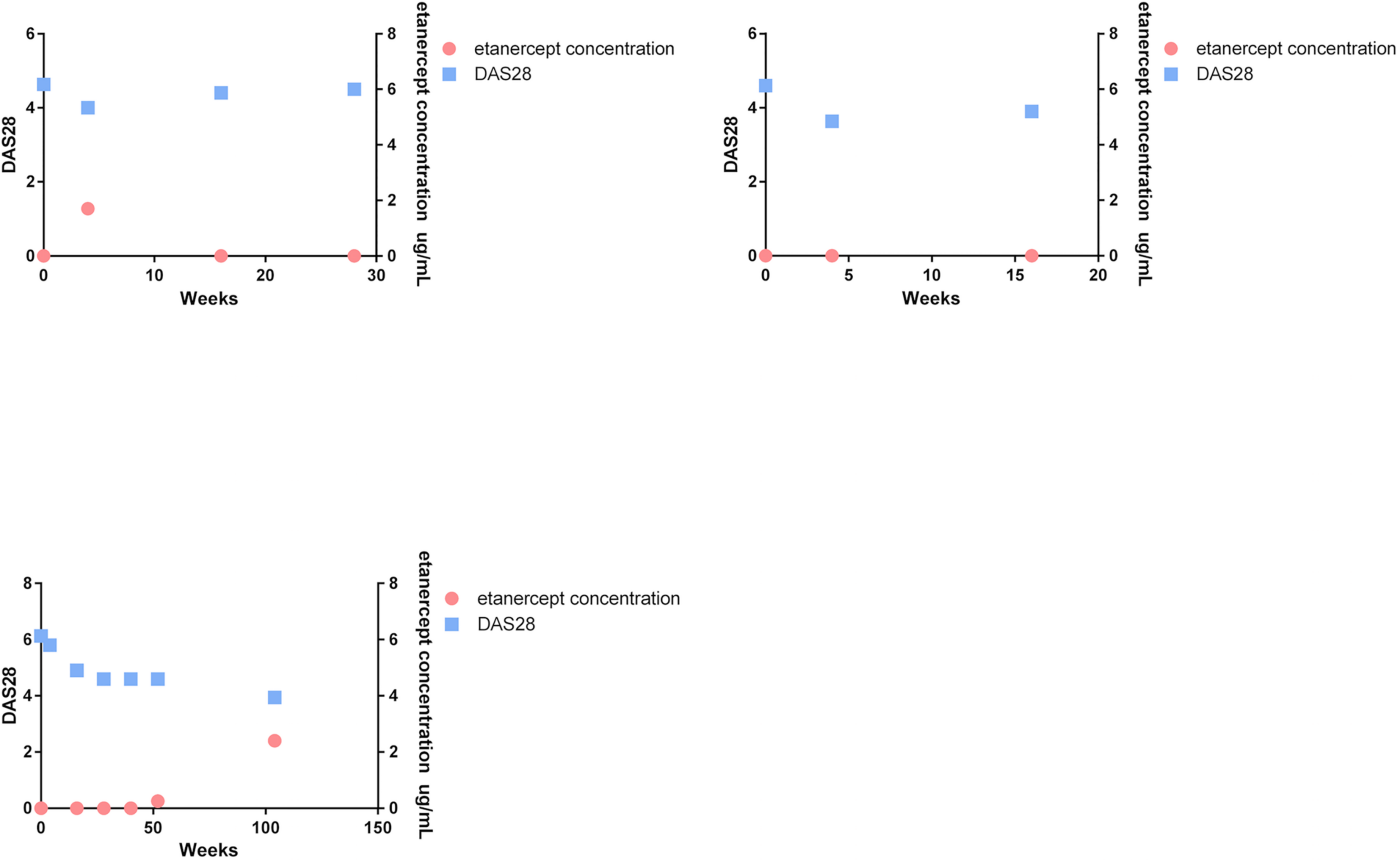
The 28 joint disease activity score (DAS28) and the etanercept concentration over time of three non-adherent patients. The DAS28 and etanercept concentration of three non-adherent patients who had an etanercept concentration <0.1μg/mL and the rheumatologist ceased etanercept treatment because of ‘treatment failure’ are individually shown. On the left Y-axis the DAS28 is depicted and on the right Y-axis the etanercept concentration (μg/mL) is shown. As seen there is relatively little change in DAS28 over time when patients had an etanercept concentration <0.1 μg/mL.

In 3 non-adherent patients, shown in Fig 2, the treating rheumatologist concluded failure of etanercept treatment (3.4% of all patients who failed treatment, 95% CI 0.8–10.4). Due to the limited number of non-adherent patients, no further analysis was done to assess the relationship between non-adherence and clinical outcome.

## Discussion

During three years of follow-up the adherence to etanercept treatment, based on etanercept concentrations and patient self-report, was high.

There is no agreed standard what constitutes ‘adequate adherence’ [17]. In our study, we defined non-adherence as follows: a serum etanercept concentration <0.1μg/mL without a valid/medical reason for missing a dose. This definition may underestimate the percentage of patients who did not take their bDMARD on the agreed date. However, the clinical relevance of a slight deviation from the standard dosing regimen remains doubtful. Nowadays, due to dose finding studies, and studies looking at tapering and stopping etanercept, it is known that a percentage of patients achieves similar clinical results on a lower dose than currently registered [18, 19]. Therefore, we argue that a slight deviation from the etanercept dosing regimen will not influence clinical outcome in a substantial percentage of patients.

The ‘white coat effect’ may result in an underestimate of the percentage of non-adherent patients [20, 21].

A strength of our study was that it is part of a large cohort study and there was no emphasis on adherence, so it reflects routine clinical practice. Furthermore, we measured serum etanercept concentrations up to a maximum of nine separate time points, providing insight into adherence behaviour over three years.

Measuring adherence through serum drug concentrations has several advantages compared with other methods. It is a direct marker whereas most other methods are indirect. Also, an etanercept concentration <0.1 μg/mL indicates that a patient is not taking the drug at the time of testing. This may be useful information for clinicians when estimating the efficacy of treatment, aiding in the provision of optimal care for patients.

One might argue that low etanercept concentrations may be caused by another mechanism, such as the development of anti-drug antibodies. Although antibodies against etanercept have been described, these were very low in frequency and of transient nature and did not influence pharmacokinetics [22, 23]. Therefore, an etanercept concentration <0.1 μg/mL can be attributed to non-adherence.

Furthermore, this study provides an interesting insight regarding immunogenicity. In adalimumab immunogenicity is associated with reduced clinical response [24]. It is generally assumed that the anti-drug antibodies are responsible for lower adalimumab concentrations and thus result in an impaired clinical response. However, this study shows that non-adherence could lead to absent drug concentrations. In the case of adalimumab if there are lower or absent drug concentrations anti-drug antibodies are more easily detected since most assays are not drug tolerant. Therefore, it could be hypothesized that in some patients non-adherence rather than anti-drug antibodies might be associated with reduced clinical response.

Our study shows that most patients missed an etanercept dose due to medical instructions provided by their treating physician, so increasing adherence in this group is not possible. Hence, when investigating adherence, methods focusing only on checking if patients have unpacked or collected their bDMARDs as prescribed seem suboptimal.

Defining adherence as “the extent to which a person’s behaviour–taking medication, following a diet, and/or executing lifestyle changes, corresponds with agreed recommendations from a health care provider” by the WHO seems more adequate and clinically relevant, but will prove more challenging when studying adherence in RA patients treated with bDMARDs [12]. Further research on adherence should therefore, at least partially, be focused on identifying patients who are non-adherent and who could potentially benefit from increased adherence. To achieve this, it is important to identify patients who temporarily stopped treatment due to medical instructions or patients in whom deviation from the dosing frequency of etanercept is possible due to low disease activity.

In conclusion, overall adherence to etanercept was high in our study, and only a small percentage of patients (4.1%) were non-adherent to etanercept therapy. In most patients missing an etanercept dose was due to a medical reason. Furthermore, this study provides insight for rheumatologists that non-adherence is low for patients being treated with etanercept and therefore that treatment failure due to non-adherence is not common.
